# Viral variant but not host factors associate with SARS-CoV-2 viral kinetics

**DOI:** 10.1038/s43856-026-01588-5

**Published:** 2026-04-17

**Authors:** William O. Hahn, Leigh H. Fisher, Amy Ward, Shannon Grant, Catherine Yen, April Kaur Randhawa, Xiaohong Li, Shelly Ramirez, Nicole Espy, John Hural, Jennifer Hanke, Pavitra Roychoudhury, Colleen F. Kelley, Nadine Rouphael, Valeria D. Cantos, Jorge Pinto, Pedro E. Cahn, Mmatsie Manentsa, Joseph Makhema, Wadzanai Samaneka, Muchaneta Bhondai-Mhuri, Lynda Stranix-Chibanda, Marcelo H. Losso, Edgar Antonio Ramirez Garcia, Craig Innes, Lameck Chinula, Javier R. Lama, Jorge A. Gallardo-Cartagena, Chishiba Kabengele, Roma Chilengi, Maphoshane Nchabeleng, Hong-Van Tieu, Brenda Hoagland, Beatriz Grinsztejn, William L. Brumskine, Khatija Ahmed, Carole L. Wallis, Alexander L. Greninger, Lawrence Corey, Ollivier Hyrien

**Affiliations:** 1 Vaccine and Infectious Disease Division, Fred Hutchinson Cancer Center, Seattle, WA USA; 2 Department of Medicine, University of Washington, Seattle, WA USA; 3 Vuka Research Clinic, University of Cape Town, Cape Town, South Africa; 4 Division of AIDS, National Institute of Allergy and Infectious Diseases, National Institutes of Health, Rockville, MD USA; 5 Division of Infectious Diseases, Department of Medicine, Emory University School of Medicine, Atlanta, GA USA; 6 Hope Clinic, Emory University, Atlanta, GA USA; 7 School of Medicine, Federal University of Minas Gerais, Belo Horizonte, Brazil; 8 Fundacion Huesped, Buenos Aires, Argentina; 9 The Aurum Institute, Johannesburg, South Africa; 10 Botswana Harvard Health Partnership, Gaborone, Botswana; 11 Department of Immunology and Infectious Diseases, Harvard T. H. Chan School of Public Health, Boston, MA USA; 12 University of Zimbabwe Clinical Trials Research Centre, Harare, Zimbabwe; 13 Faculty of Medicine and Health Sciences, University of Zimbabwe, Harare, Zimbabwe; 14 Emerging Diseases Research Unit, Hospital JM Ramos Mejia, Buenos Aires, Argentina; 15 Asociación Civil Selva Amazónica CRS, Universidad Nacional de la Amazonia Peruana, Iquitos, Peru; 16 Division of Global Women’s Health, Department of Obstetrics and Gynecology, University of North Carolina at Chapel Hill, Chapel Hill, NC USA; 17 Asociacion Civil Impacta Salud y Educacion, Lima, Peru; 18 Universidad Nacional Mayor de San Marcos, Centro de Investigaciones Tecnológicas, Biomédicas y Medioambientales, Lima, Peru; 19 Center for Family Health Research in Zambia, Lusaka, Zambia; 20 Zambia National Public Health Institute, Ministry of Health, Lusaka, Zambia; 21 Mecru Clinical Research Unit, Sefako Makgatho Health Sciences University/National Health Laboratory Service, Dr George Mukhari Tertiary Microbiology Laboratory, Pretoria, South Africa; 22 Lindsley F. Kimball Research Institute, New York Blood Center, New York, NY USA; 23 Division of Infectious Diseases, Department of Medicine, Vagelos College of Physicians and Surgeons, New York-Presbyterian Columbia University Irving Medical Center, New York, NY USA; 24 Instituto Nacional de Infectologia Evandro Chagas, Fundação Oswaldo Cruz (INI-Fiocruz), Rio de Janeiro, Brazil; 25 The Aurum Institute NPC, Johannesburg, South Africa; 26 Department of Infectious Disease, Vanderbilt University School of Medicine, Nashville, TN USA; 27 Setshaba Research Centre, Soshanguve, South Africa; 28 Department of Medical Microbiology, School of Medicine, Faculty of Health Sciences, University of Pretoria, Pretoria, South Africa; 29 Department of Paediatrics & Child Health, in the School of Clinical Medicine, University of the Witwatersrand, Johannesburg, South Africa; 30 BARC-SA and Lancet Laboratories, Johannesburg, South Africa; 31 Department of Laboratory Medicine and Pathology, University of Washington Medical Center, Seattle, WA USA; 32 Department of Laboratory Medicine and Pathology, University of Washington, Seattle, WA USA

**Keywords:** Viral infection, Public health

## Abstract

**Background:**

It is challenging to assess SARS-CoV-2 viral kinetics amidst viral variant evolution and changes in population-level immunity. However, understanding the relationship between host factors and viral replication deepens our understanding of viral fitness.

**Methods:**

In CoVPN 5001, we enrolled *N* = 953 adults diagnosed with acute SARS-CoV-2 from July 2020 to July 2022 across 51 sites. We confirmed SARS-CoV-2 infection by RT-PCR and identified the variant via viral genome sequencing. Using multivariable linear regression and median regression, we studied the association between host factors and either observed peak viral load (VL) or clinical viral shedding, respectively, accounting for viral variant effects in a demographically and clinically diverse longitudinal cohort.

**Results:**

In this observational study, we determine that while host factors, including age, BMI, sex, medical comorbidities, and HIV, have no significant associations with either observed peak VL or clinical viral shedding in the nasopharynx, viral variant is significantly associated with observed peak VL and clinical viral shedding. We show that neither observed peak VL nor shedding duration predict evolutionary success since the dominant variants in the SARS-CoV-2 pandemic did not all align with the variants with the highest peak and longest shedding duration.

**Conclusions:**

Altogether, our work shows that observed peak VL and shedding duration should be cautiously interpreted as predictors of viral fitness.

## Plain language summary

Over time the virus causing COVID has changed, with these different viruses described as viral variants that have started in specific geographic regions. Many studies of COVID have involved small numbers of people infected with only one or two viral variants. We undertook a study of a large number of people across the USA, Southern Africa, and South America who acquired COVID between 2020 and 2022, a period during which many different viral variants were present. Different viral variants resulted in different amounts of virus being present in the upper-airways. However, a large amount of virus being present did not predict that a variant would outcompete other variants. Better understanding of the impact of viral variants offers potential improvements for public health policy and insight into the COVID pandemic to date.

## Abstract (web summary)

Hahn et al. use a diverse clinical cohort to test for associations of clinical variables with SARS-CoV-2 kinetics. They find minimal associations with known clinical variables but do find that different viral strains are associated with different viral kinetics.

## Introduction

The effects of host demographic and clinical factors on viral replication during clinical SARS-CoV-2 infection are still poorly understood. Despite the plausible link between host factors and both viral load (VL) and time to viral clearance, investigations into this relationship during clinical infection have resulted in conflicting findings^1–4^. Understanding viral kinetics during clinical infection is challenging because the COVID-19 pandemic has been characterized by a series of emerging viral variants replacing previously circulating strains, with different replication kinetics among variants and population-level immunity to these variants changing over time^5^. Therefore, studies of the association between host demographics and viral kinetics should account for potential variant effects and would ideally involve a demographically and clinically diverse longitudinal cohort recruited throughout the pandemic.

Some studies comparing the viral kinetics of variant strains have suggested marked increases in viral burden with newly emerging variants, potentially related to immune escape^6–9^. However, such differences in viral kinetics between strains have not been universally observed, as other groups have found no differences in the viral burden across different variants^10–12^. Firm conclusions regarding the replication kinetics of variants in humans have been complicated by differences in sampling, lack of generalizability due to limited variant comparisons (e.g., only comparing Delta versus Alpha), non-representative populations, small sample sizes, and a lack of longitudinal follow-up^9^.

The relationship between host demographic factors, comorbidities, and readily measurable virologic features, such as VL and shedding duration, has implications for the pathophysiology of COVID-19 disease. Higher viral burden and failure to clear virus in patients have been associated with poorer clinical outcomes, hypothesized to be related to the production of inflammatory cytokines such as IL-6^1,13,14^. Additionally, while there is some heterogeneity across studies, generally reduction in VL via pharmacologic intervention in unvaccinated populations is associated with improved clinical outcomes^15^, and VL and shedding duration measured via quantitative PCR tracked throughout the pandemic have demonstrated that an absence of detectable virus in the nasopharynx makes transmission extremely unlikely^6^. Studies have found associations between VL and risk of transmission^16^. In addition, understanding how viral kinetics have shifted throughout the pandemic could inform future predictive models and policy-making during emerging viral pandemics.

To understand the associations of both viral variant and host demographic factors with the kinetics of viral replication during clinical infection, we present the first analysis of the CoVPN 5001 study, a large longitudinal cohort that recruited 953 participants from over 50 global sites across three geographic regions (Southern Africa, Latin America, and the United States of America), spanning the spectrum of COVID-19 disease, and including viral variants ranging from the reference (pre-Alpha) to the early Omicron strains during July 2020 through March 2022. Using this virologically and clinically diverse cohort, we investigated associations of host demographics and viral variants with viral kinetics.

We determine that host characteristics and clinical variables exhibited minimal associations with both observed peak VL (i.e., the practical maximum observed VL in a clinical trial where enrollment occurs after the onset of symptoms) and clinical viral shedding duration (the practical time of viral shedding after diagnosis), suggesting that these variables may not be useful in tailoring public health interventions targeting populations with potentially higher transmission risks. Our analysis shows that variants responsible for infection associate with differences in observed peak VL and shedding duration. Importantly, neither observed peak VL nor the shedding duration predict evolutionary success since the dominant variants in the SARS-CoV-2 pandemic did not all align with the variants with the highest observed peak VL and longest shedding duration. The clinical and epidemiological implications of our findings are that observed peak VL and shedding duration should be interpreted cautiously, especially as new SARS-CoV-2 variants continue to emerge, and that host characteristics are unlikely to cause poor clinical outcomes solely via alterations in VL.

## Methods

### Study design

We conducted an observational study involving 953 adult individuals diagnosed with acute SARS-CoV-2 infection, with enrollment spanning from July 2020 through March 2022 across 51 sites located in 10 countries: Botswana, Malawi, South Africa, Zambia, Zimbabwe in Southern Africa; USA in North America; Mexico, Argentina, Brazil, Peru in Latin America. The study was extended to capture the emergence of the Omicron cohort.

Eligibility criteria for enrollment included individuals aged ≥18 years who reported a positive SARS-CoV-2 test result from assays authorized for emergency use by the US Food and Drug Administration or approved as standard of care by other relevant regional regulatory bodies. Participants were stratified based on their clinical presentation into three categories: those who were asymptomatic with a positive test within 10 days before enrollment; individuals with symptom onset within 14 days before enrollment not requiring hospitalization, and a positive test within 10 days prior to enrollment; and individuals with symptoms requiring hospitalization within 3 days before enrollment.

Standardized case report forms were used to collect demographic data and clinical information for each participant. Nasal or nasopharyngeal swabs for quantitative SARS-CoV-2 real-time reverse transcription polymerase chain reaction (RT-PCR) and viral sequencing were collected at enrollment and on study days 2, 7, 14, 21, and 28.

The study was designed to detect significant differences when comparing continuous variables (e.g., VL) measured in two independent groups of varying sizes using a two-sided Wilcoxon rank-sum test with a 5% significance level. For example, the study achieves >80% power assuming two independent, normally distributed populations of respective sizes *n*1 = 80 and *n*2 = 60 and a standardized (Cohen’s) effect size of 0.50.

### Ethics

All participants provided written informed consent. For clinical research sites in the US, the study was reviewed and approved by a single institutional review board (IRB), Advarra (Columbia, MD). For sites outside the US, the study was reviewed and approved by all relevant regulatory bodies and ethics committees. The trial was registered at clinicaltrials.gov (NCT04431414). IRBs for the sites are listed in the Supplementary Data.

### Viral detection

Nasal and nasopharyngeal swabs were stored in universal or viral transport media and frozen until testing. SARS-CoV-2 VL was quantified in Southern Africa by comparing the TaqPath™ COVID‑19 CE‑IVD RT‑PCR Assay (ThermoFisher, Waltham, MA, USA) S, N, and Orf gene cycle thresholds (Ct) to a standard curve. VLs in the Americas were obtained from Cobas® SARS-CoV-2 RT-PCR Test (Roche, Indianapolis, IN, USA) wherein the E and Orf1ab gene cycle thresholds compared to a standard curve^17^. VLs (copies/mL) were averaged across detected targets and log_10_ average VL is reported.

To ensure that VL measurements were consistent across labs, assay standards were run concurrently for conversion to copies/mL. Additionally, a subset of samples was selected and aliquots were sent to each lab; results from the two labs were found to be consistent (Supplementary Fig. 1).

### Viral sequencing

Viral genome sequencing was performed to identify the SARS-CoV-2 variant each study participant acquired. The swab with the highest VL (or lowest Ct) that met the sequencing criteria was selected for viral genome sequencing for each individual. For samples run on TaqPath, sequencing was considered if the VL for Orf1ab was at least 400 copies/mL, or an average VL of N and Orf1ab was at least 500 copies/mL. For samples run on Cobas, sequencing was considered if the Ct for E-gene was less than 35. If the initial swab from a given participant failed to be sequenced, the next highest VL swab meeting the criteria was attempted. RNA was extracted from residual clinical specimens using either Roche MagNA Pure 96 or ThermoFisher KingFisher platforms following the manufacturer’s instructions. SARS-CoV-2 whole genome sequencing was performed using the Swift Normalase Amplicon Panel (xGen SNAP, Integrated DNA Technologies) protocol automated on the Sciclone G3 NGSx iQ liquid handling system (Revvity) and sequenced on Illumina NextSeq 2000 instruments using a 2 × 150 bp read format^18^. Raw reads were processed using a custom bioinformatics pipeline (https://github.com/greninger-lab/covid_swift_pipeline)^19^. Viral lineages were assigned to sequences using Phylogenetic Assignment of Named Global Outbreak Lineages (PANGOLIN). Named variants were matched to the WHO-defined variant labels. Reference is defined as the basal outbreak lineage B.1, which bears the D614G mutation^20^.

### Statistical methods

The primary aim of the statistical analysis was to study the association between demographic, clinical factors, and SARS-CoV-2 variants with observed peak VL and viral clearance. Participants were classified into three categories according to COVID-19 severity at enrollment: asymptomatic, mild symptoms not requiring hospitalization, and hospitalized. Analyses were stratified by region (Southern Africa, Latin America, and the USA) to account for regional variations attributed to host genetics and environmental factors. Mexico was included with South American countries due to similar regional dissemination patterns of COVID variants^21^, such as the Lambda variant. First COVID Day was defined as the day of earliest symptom onset or first positive PCR test, whichever occurred first. “COVID Days” was defined as the number of days since the earliest symptom onset or first positive PCR test, whichever occurred first. Shedding duration was defined as COVID Days until viral clearance, defined as the first instance of two consecutive PCR-negative swabs. Observed peak VL was defined as the highest VL observed during the study.

We used multivariable linear regression to explore the relationship between observed peak log_10_(VL) while on study and various covariates, including geographical region, COVID-19 severity at enrollment, COVID Days at enrollment, age (in years), age squared (to account for potential non-linear relationships), body mass index (BMI), sex, presence (yes/no) of human immunodeficiency virus (HIV), diabetes, hypertension, chronic kidney disease, cancer, and immune system disorder, presence of any comorbidity (yes/no), smoking status (self-reported tobacco and/or marijuana), COVID-19 vaccination and its interaction with the time elapsed since the last COVID-19 vaccine, and the acquired SARS-CoV-2 variant.

We studied the association between clinical viral shedding duration (in days) and covariates using median regression (50th quantile regression), an alternative to standard regression that is robust against outliers. Because VL was measured at 6 time points over ~4 weeks, the duration of clinical viral shedding could not be exactly observed and was treated as interval censored in the analysis. Quantile regression for interval censored data has been used in previous investigations of SARS-CoV-2 shedding^22^. The median regression model adjusted for the same set of variables as the multivariable linear regression for peak log_10_(VL), except for COVID day at enrollment.

The variant-specific cumulative distribution function (CDF) of shedding duration was estimated using quantile regression, applying multiple imputation to handle missing viral variants. To visualize the corresponding density functions as violin plots stratified by viral variant, data were simulated from the estimated CDF via inverse transform sampling.

We used generalized additive models (GAM) to describe the association between peak log_10_(VL) and calendar time due to its flexibility in allowing smoothed nonlinear relationship between these variables. Similar analyses were carried out to examine the association between observed peak VL and age (in years), observed peak VL and BMI, COVID Days at enrollment and calendar time. In these analyses, the model adjusted for geographical region and handled missing viral variants through multiple imputation. The reference group was defined as asymptomatic female participants in Southern Africa without comorbidities who acquired the reference variant.

Statistical analyses addressed missing viral variants using multiple imputation whereby missing variants were randomly imputed based on a variant predictive model built using a GAM that included the geographical location and calendar time at which participants enrolled in the study as predictors. The model also included a smooth function of calendar time to allow non-linear temporal variation in the frequency of each variant within each region. The model was trained using public surveillance data from the GISAID database. Standard errors of regression coefficients were estimated using Rubin’s rules. Model predictive performances achieved more than 90% accuracy when applied to predict the viral variant CoVPN 5001 acquired by CoVPN 5001 participants for whom the actual acquired SARS-CoV-2 variant was successfully identified via sequencing. This excellent performance was attributed to the rapid transition in viral dominance and virtually complete replacement of variants between successive waves. No further refinement of the predictions of viral variants with baseline covariates and ensemble learning could be achieved.

All hypothesis tests were two sided. *P*-values were adjusted for multiple comparisons by controlling the false discovery rate (FDR) using the Benjamini-Hochberg procedure. All statistical analyses were conducted using R version 4.1.3.

## Results

### Participant demographics and clinical factors

During July 2020 through March 2022, the study enrolled 953 participants in three geographical regions—USA (11%), Latin America (44%), and Southern Africa (45%). Of these participants, 98 (10.3%) were asymptomatic at enrollment, 815 (85.5%) were symptomatic and not hospitalized, and 40 (4.2%) were hospitalized (Table 1). The median age of participants was 37 years; 18% were aged >50 years. Sex was comparable across regions and the overall cohort was 53% female. Of all participants, 27.1% reported at least one comorbidity, with the most common being hypertension (14%), current smoking (11%), HIV (8%), and diabetes (5%) (Supplemental Table 1). 19.5% of the participants had a BMI ranging between 30 and 35 (obese), 9.5% between 35 and 40 (severely obese), and 3.9% had a BMI exceeding 40. History of SARS-CoV-2 vaccination prior to enrollment was reported by 16% of participants. Vaccines received included inactivated vaccines (*n* = 26), mRNA vaccines (*n* = 33), inactivated viral vector vaccines (*n* = 9), and combinations of products (*n* = 7). Ongoing systemic corticosteroids (excluding short course of dexamethasone) were reported at study entry in 10.6% of participants, whereas short-course dexamethasone was reported in 5.0% of participants (Supplemental Table 1).

**Table 1:** CoVPN 5001 participant demographics

Characteristic		Cohort			Total 953
		Group 1: Asymptomatic 98 (10.3%)	Group 2: Mild symptoms, not hospitalized 815 (85.5%)	Group 3: Hospitalized 40 (4.2%)	
Region	Southern Africa	75 (76.5%)	339 (41.6%)	13 (32.5%)	427 (44.8%)
	Latin America	12 (12.2%)	385 (47.2%)	26 (65.0%)	423 (44.4%)
	United States of America	11 (11.2%)	91 (11.2%)	1 (2.5%)	103 (10.8%)
Country	Argentina	0 (0.0%)	21 (2.6%)	10 (25.0%)	31 (3.3%)
	Botswana	9 (9.2%)	17 (2.1%)	0 (0.0%)	26 (2.7%)
	Brazil	4 (4.1%)	45 (5.5%)	11 (27.5%)	60 (6.3%)
	Malawi	6 (6.1%)	12 (1.5%)	0 (0.0%)	18 (1.9%)
	Mexico	0 (0.0%)	7 (0.9%)	0 (0.0%)	7 (0.7%)
	Peru	8 (8.2%)	312 (38.3%)	5 (12.5%)	325 (34.1%)
	South Africa	47 (48.0%)	205 (25.2%)	7 (17.5%)	259 (27.2%)
	USA	11 (11.2%)	91 (11.2%)	1 (2.5%)	103 (10.8%)
	Zambia	3 (3.1%)	67 (8.2%)	4 (10.0%)	74 (7.8%)
	Zimbabwe	10 (10.2%)	38 (4.7%)	2 (5.0%)	50 (5.2%)
Sex	Female	48 (49.0%)	440 (54.0%)	15 (37.5%)	503 (52.8%)
	Male	50 (51.0%)	375 (46.0%)	25 (62.5%)	450 (47.2%)
Age	Mean (SD)	38.7 (13.18)	38.4 (12.54)	47.3 (12.78)	38.8 (12.72)
	Medium (25th, 75th %ile)	38.5 (28.0, 48.0)	37.0 (28.0, 47.0)	46.5 (37.5, 58.5)	37.0 (28.0, 47.0)
	Min–Max	18.0–70.0	18.0–87.0	20.0–76.0	18.0 - 87.0
Age group	18–50	77 (78.6%)	678 (83.2%)	23 (57.5%)	778 (81.6%)
	Over 50	21 (21.4%)	137 (16.8%)	17 (42.5%)	175 (18.4%)
Baseline BMI (kg/m^2^)^a^	Not reported	2 (2.0%)	10 (1.2%)	2 (5.0%)	14 (1.5%)
	Underweight <18.5 kg/m^2^	8 (8.2%)	11 (1.3%)	1 (2.5%)	20 (2.1%)
	Normal weight 18.5–24.9 kg/m^2^	32 (32.7%)	239 (29.3%)	3 (7.5%)	274 (28.8%)
	Overweight 25–29.9 kg/m^2^	23 (23.5%)	292 (35.8%)	16 (40.0%)	331 (34.7%)
	Obese 30–34.9 kg/m^2^	17 (17.3%)	158 (19.4%)	11 (27.5%)	186 (19.5%)
	Severely Obese 35–39.9 kg/m^2^	14 (14.3%)	75 (9.2%)	2 (5.0%)	91 (9.5%)
	Extremely Obese ≥40 kg/m^2^	2 (2.0%)	30 (3.7%)	5 (12.5%)	37 (3.9%)
Medical comorbidities	No	71 (72.4%)	603 (74.0%)	21 (52.5%)	695 (72.9%)
	Yes	27 (27.6%)	212 (26.0%)	19 (47.5%)	258 (27.1%)

^a^BMI as defined by World Health Organization (WHO).

### Associations between host factors, clinical variables, and viral factors and observed peak viral load

**Fig. 1:**
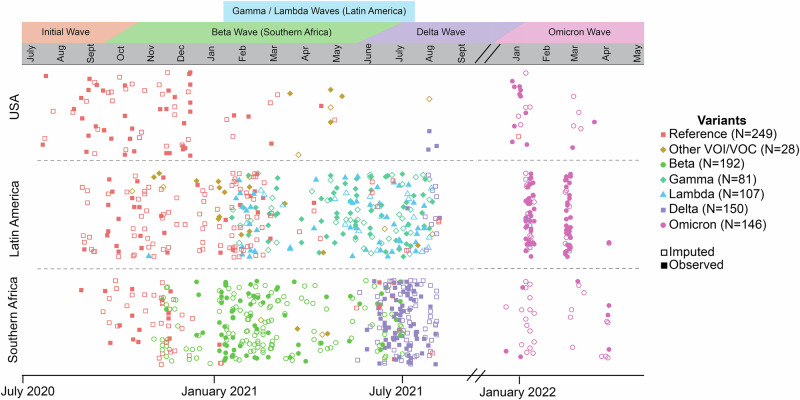
The temporal dynamics of variant spectrums identified via PCR sequencing in nasal specimens from CoVPN 5001 (*N* = 953), stratified by region. Cases confirmed by sequencing are shown in closed symbols, whereas imputed cases are shown as open symbols. The break between July 2021 and January 2022 represents a period where the protocol was not actively enrolling and there are no observations. “Other VOI/VOC” includes the Alpha, Iota, Mu, and Zeta variants.

**Fig. 2:**
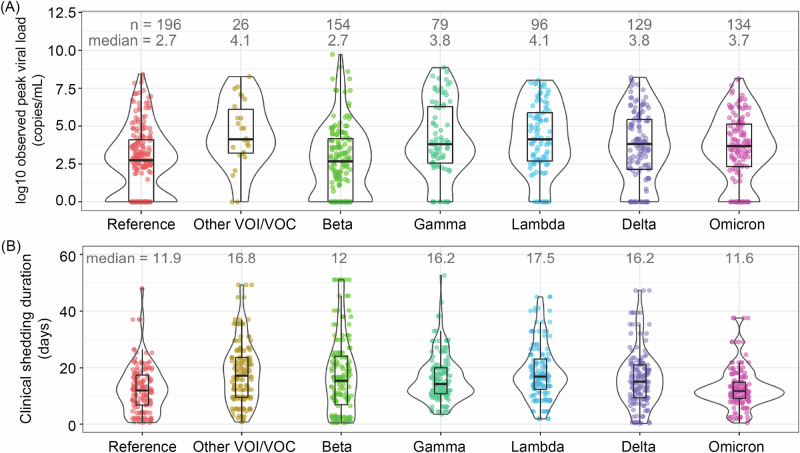
Observed peak viral load and clinical shedding duration (*N* = 814) by SARS-CoV-2 variant. **A** Observed peak viral load on study in copies/mL in participants with mild symptoms, stratified by variant. **B** Duration of shedding in participants who acquired the respective variant. Shedding duration is defined as the first of two measurements with no detectable virus. The box represents 25% and 75% percentile. The whiskers extend to the smallest and largest values within 1.5 times the interquartile range (IQR) from the first and third quartiles, respectively.

The viral variants reported in our cohort are plotted over calendar time in Fig. 1. A total of 249 (26%) participants had confirmed or predicted acquisition of the reference strain (the basal outbreak lineage B.1, which bears the D614G mutation). In addition, the following variants of concern (VOC) or variants of interest (VOI) were detected in the cohort: Beta (*n* = 192, 20%), Gamma (*n* = 81, 9%), Lambda (*n* = 107, 11%), Delta (*n* = 150, 16%), and Omicron (*n* = 146, 15%) (Fig. 1). Almost all Beta acquisitions were isolated from Southern Africa, whereas the Gamma and Lambda acquisitions were isolated in Peru and Brazil. Other participants (*n* = 28, 3%) acquired other VOI/VOC, for example, Alpha, all of which appeared in the early stage of the pandemic but had limited representation in the cohort. In our analysis, we grouped these lineages into a common category referred to as *Other VOC/VOI*. In symptomatic, non-hospitalized participants, we observed differences in both the observed peak VL and shedding duration between viral variants (Fig. 2), with both observed peak VL and shedding duration appearing smaller in the Reference variant compared to subsequent variants until the Omicron variant. Observed peak VL and viral clearance may be impacted by different host factors. For example, age has different effects on the innate versus adaptive immune systems during SARS-CoV-2 infection^23^. Therefore, we performed two separate multivariate regression analyses of VL and clinical viral shedding duration adjusting for both demographic, clinical, and viral factors to account for potential confounders. This includes adjustment for day of symptom onset.

We first investigated whether host factors generally accepted to be associated with clinical outcomes were also associated with observed peak VL (Table 2). We determined that the presence of clinical symptoms (in mild or hospitalized participants) associated with higher observed peak VLs compared to asymptomatic participants (adjusted *p*-value < 0.001 for both symptomatic and hospitalized participants, estimated around 2 log higher in both groups). In contrast, demographic and clinical factors, including age, BMI, sex, hypertension, chronic kidney disease, cancer, diseases of the immune system (such as rheumatologic diseases), diabetes, active smoking, and living with HIV were not associated with observed peak VL (*p*-value > 0.05). As expected, a longer interval between COVID day and day of enrollment in the study was also associated with a decrease in observed peak VL (*p*-value < 0.001), with an average decrease of −0.20 log per additional day between COVID day and enrollment.

**Table 2:** Multivariable regression analysis of observed peak viral load

Clinical Variable		Coefficient	Adjusted *p*-value	*p*-value	95% CI
Intercept		3.61	1.70 × 10^−5^****	3.26 × 10^−6^****	2.09–5.14
Disease severity at enrollment	(ref. = asymptomatic)				
Mild symptoms		1.72	2.57 × 10^−10^****	1.98 × 10^−11^****	1.22–2.23
Hospitalized		2.38	3.74 × 10^−6^****	4.32 × 10^−7^****	1.46–3.30
Region	(ref. = Southern Africa)				
Latin America		0.12	0.77	0.68	−0.41–0.67
United States of America		0.78	0.060^#^	0.021*	0.12–1.43
Any comorbidity	(reference = no)	0.16	0.77	0.68	−0.61–0.94
Age (years)		−0.051	0.22	0.12	−0.12– 0.014
Age^2^		0.00066	0.20	0.083^#^	−0.000087–0.0014
BMI		−0.0054	0.77	0.68	−0.031– 0.020
Sex	(ref. = female)	−0.027	0.90	0.86	−0.32–0.27
Diabetes	(ref. = no)	0.066	0.90	0.87	−0.70–0.83
Active smoking	(ref. = no)	−0.25	0.39	0.29	−0.73–0.22
HIV	(ref. = PWOH)	−1.05	0.22	0.11	−2.33–0.23
Hypertension	(ref. = no)	0.15	0.77	0.68	−0.58– 0.89
Chronic kidney disease	(ref. = no)	−0.025	0.98	0.98	−1.72– 1.67
Cancer	(ref. = no)	−0.57	0.33	0.20	−1.44–0.31
Immune system disease	(ref. = no)	0.99	0.22	0.12	−0.27–2.26
COVID-19 vaccination	(ref. = no)	0.51	0.39	0.28	−0.41–1.43
COVID-19 vaccination × Time since last vaccination		−0.0045	0.21	0.097	−0.0099– 0.00082
COVID day		−0.20	1.35 × 10^−23^****	5.19 × 10^−25^****	−0.24–0.17
**Viral variant**	**(ref. = Reference)**	**Coefficient**	**Adjusted *p-*value**	***p-*value**	**95% CI**
Other VOI/VOC		1.31	0.046*	0.014*	0.27–2.36
Beta		0.62	0.15	0.056	−0.017 – 1.26
Gamma		1.48	8.10 × 10^−5^****	1.87 × 10^−5^****	0.80–2.16
Lambda		1.50	1.29 × 10^−5^****	1.98×10^−6^****	0.88–2.12
Delta		1.02	0.0056**	0.0015**	0.39–1.64
Omicron		0.57	0.33	0.21	−0.33–1.47

Bolded variables indicate those that are statistically significant. # indicates variables trending toward statistical significance. *Age*^*2*^ is included to account for a potential non-linear relationship. Analysis was two-sided. *COVID-19 vaccination x Time since last vaccination* indicates an interaction. For those who did not receive vaccination, *Time since last vaccination* is set at an undefined default value (conceptually at 0). The choice of this default value has no impact on the analysis. ** indicates *p* ≤ 0.01; *** indicates *p* ≤ 0.001; **** indicates *p* ≤ 0.0001

Our analysis also investigated the association between viral variant and observed peak VL (Table 2). All variants after the Reference variant had higher point estimates of observed peak VL when compared to the Reference variant. In addition, after correcting for multiple comparisons, we determined that all VOC and/or VOI present in our cohort had higher average peak log VLs when compared to the reference strain (adjusted *p*-value < 0.05), except for the Beta and Omicron variants (adjusted *p*-value = 0.15 and 0.33, respectively). Compared to the reference strain, the magnitude of increase of the peak log VL from the Beta variant (increase of log 0.62 copies/mL) to the Delta variant wave transition (increase of log 1 copies/mL) in Southern Africa followed the epidemiology of SARS-CoV-2 strains, where the Beta variant rapidly supplanted the reference strain and was, in turn, rapidly replaced by the Delta variant (Fig. 1). This pattern did not hold in other regions, as the magnitude of the increase compared to the reference strain was higher for the Gamma and Lambda variants (increase of 1.48 and 1.50 log copies/mL), both of which were ultimately outcompeted by the Delta variant in Latin America. Although circulating SARS-CoV-2 variants varied between regions, there was no difference between the Southern Africa and Latin America regions after adjusting for variant (adjusted *p*-value = 0.78). This finding suggests that regional variations in observed peak VL largely reflected differences in circulating strains. As VL from the Southern Africa region was quantified using a different machine than the Latin America and USA regions, our data suggest that the method of quantification did not affect our results. Region may be a residual source of variation, however, as we did observe a potential difference between the observed peak VL in the United States compared to Southern Africa (estimated increase of 0.7 log copies/mL; adjusted *p*-value = 0.093).

### Associations between host factors, clinical variables, viral factors and duration of clinical viral shedding

Observed peak VL may not fully capture viral fitness during clinical infection as the duration of viral persistence could also influence viral spread. Therefore, we performed a separate multivariable regression analysis to assess whether any of the demographic, clinical, and viral variant factors used in the previous analysis were associated with overall duration of clinical viral shedding (Table 3). Like our findings for observed peak VL, we found that age, BMI, sex, hypertension, chronic kidney disease, cancer, diseases of the immune system (such as rheumatologic diseases), diabetes, smoking, and HIV were not associated with the shedding duration (adjusted *p*-values ≥0.25). We determined that participants with mild symptoms not requiring hospitalization or hospitalized cases were associated with longer shedding than asymptomatic cases (adjusted *p*-values < 0.001). No significant association was found between COVID-19 vaccination and observed peak VL (adjusted *p*-value ≥0.25).

**Table 3:** Multivariable regression analysis of clinical viral shedding duration

Clinical Variable		Coefficient	Adjusted *p*-value	*p*-value	95% CI
Intercept		7.20	0.25	0.073	−0.6–615.06
Disease severity at enrollment	(ref. = asymptomatic)				
Mild symptoms		10.80	3.55 × 10^−13^****	1.48 × 10^−14^****	8.05–13.56
Hospitalized		14.11	4.36 × 10^−11^****	3.63 × 10^−12^****	10.13–18.09
Region	(ref. = Southern Africa)				
Latin America		−0.30	0.86	0.82	−2.90–2.31
United States of America		2.61	0.25	0.090	−0.41–5.62
Any comorbidity	(reference = no)	−0.90	0.84	0.67	−4.99–3.19
Age (years)		−0.27	0.25	0.11	−0.61–0.057
Age^2^		0.0033	0.25	0.10	−0.00065– 0.0072
BMI		−0.039	0.75	0.53	−0.16– 0.082
Sex	(ref. = female)	−0.20	0.84	0.77	−1.57–1.18
Diabetes	(ref. = no)	2.68	0.51	0.25	−1.91–7.28
Active smoking	(ref. = no)	−0.44	0.84	0.68	−2.54–1.65
HIV	(ref. = PWOH)	1.17	0.75	0.76	−53.31–39.20
Hypertension	(ref. = no)	−7.06	0.84	0.53	−2.51–4.85
Chronic kidney disease	(ref. = no)	−5.13	0.57	0.31	−15.05–4.78
Cancer	(ref. = no)	0.41	0.87	0.87	−4.38–5.20
Immune system disease	(ref. = no)	7.41	0.84	0.75	−38.53–53.36
COVID-19 vaccination	(ref. = no)	−1.59	0.75	0.44	−5.61–2.43
**Viral variable**	**(ref. = Reference)**	**Coefficient**	**Adjusted*p-*value**	***p-*value**	**95% CI**
Other VOI/VOC		6.39	0.14	0.031*	0.60–12.17
Beta		2.69	0.41	0.19	−1.30–6.69
Gamma		3.60	0.14	0.036*	0.23–6.95
Lambda		6.24	6.80 × 10^−4^***	8.50 × 10^−5^****	3.13–9.35
Delta		6.04	0.0026**	0.00044***	2.67–9.41
Omicron		1.41	0.75	0.50	−2.72–5.50

**indicates *p* ≤ 0.01; ***indicates *p* ≤ 0.001; ****indicates *p* ≤ 0.0001

Clinical viral shedding duration in the reference group was estimated to have a median of 7.2 days for asymptomatic cases caused by the Reference strain in  those assigned female at birth (AFAB), living in Southern Africa and without any comorbidities (Table 3). Median shedding duration was estimated at 18.0 days (Reference estimate of 7.2 days, with the additional 10.8 days) for symptomatic participants and 21.3 days for hospitalized participants. Our analysis indicated that shedding duration was longer for all other viral strains compared to the Reference variant in the analysis unadjusted for multiple comparisons (Supplemental Table 3). Only the Lambda and Delta variants remained significant after adjusting for multiple comparisons, with increased shedding duration of 6.2 days for Lambda (adjusted *p*-value < 0.001), and 6.0 days for the Delta variant (adjusted *p*-value = 0.0026). One notable exception was the Beta variant, for which median shedding duration was not found to be significantly longer compared to the Reference strain (an additional 2.7 days; adjusted *p*-value = 0.41). The Omicron variant was associated with an increase of shedding duration by 1.4 days compared to the Reference variant, without reaching statistical significance (adjusted *p*-value = 0.75).

### Fluctuations in observed peak VL

**Fig. 3:**
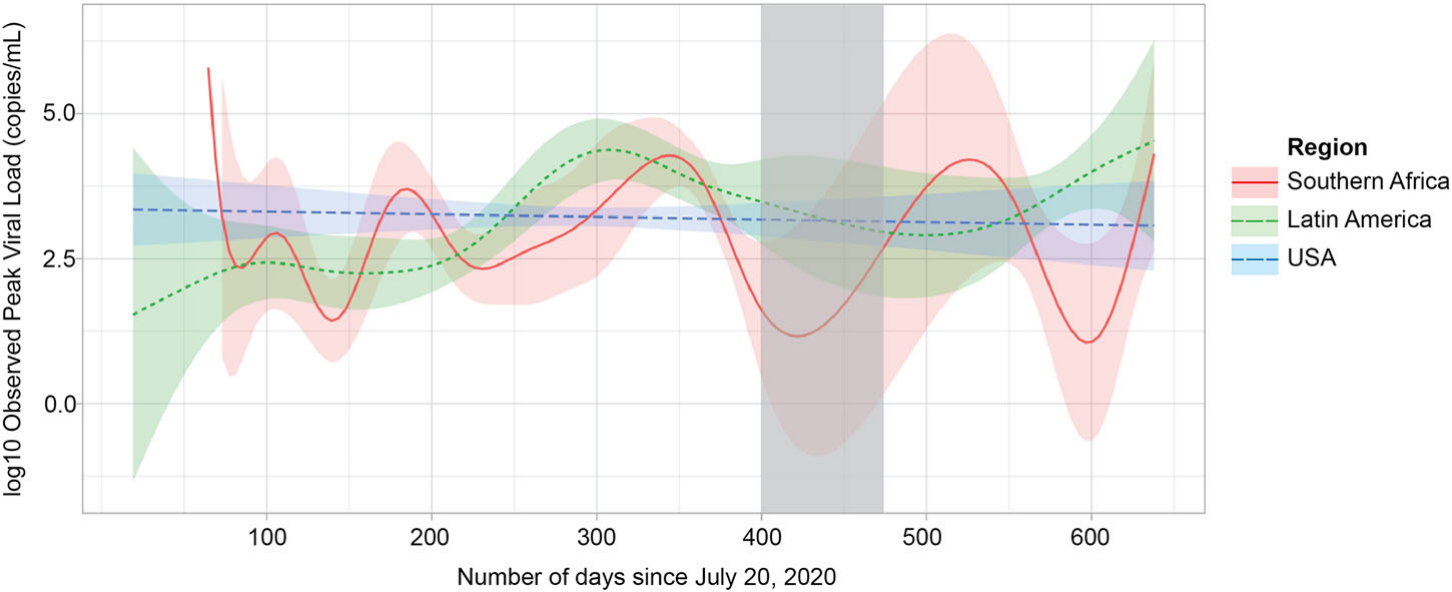
Dynamics of the average log_10_ observed peak viral load (in copies/mL) over time stratified by region, as estimated by general additive modelling (GAM) (*N* = 953). The shaded area associated with each region indicates the point-wise 95% confidence interval for average log_10_ observed peak viral load. The gray box represents a period where the protocol was not actively enrolling and there are no observations. Day zero represents July 20, 2020. The time period extends until the date of enrollment of the final participant.

Given that observed peak VL and clinical viral shedding duration were associated with some variant types, we hypothesized that the observed peak VL might have changed as the pandemic progressed, either increasing due to gradual viral adaptation to the host, or decreasing due to stronger immunologic memory, or a combination of both scenarios. We observed substantial fluctuation in the observed peak VL over time (Fig. 3). If observed peak VL was driven solely by the infecting variant, we would expect changes in VL to mirror changes in the infecting variant. For example, we would predict that the highest VL in our study would coincide with the time period where the majority of cases were caused by the Delta variant. However, we did not observe this pattern (Supplementary Fig. 2).

We observed that observed peak VL oscillated over time and differed by region (Fig. 3 and Supplementary Fig. 2). Previous investigations had determined that VLs increased during “wave” periods^24^. Some time periods where VL appeared to increase coincided with the emergence of new variants in specific regions, consistent with this observation^24^. For example, our GAM analysis detected significant non-linear temporal variations in observed peak VL in Latin America and Southern Africa (*p*-values < 0.001) but not in the USA (*p*-value = 0.57). It also suggested that in Southern Africa, observed peak VL decreased between July and October 2020, ~100 days after the study began. This period coincided with the initial appearance of the Beta variant in the region. As the Beta variant became more prevalent, observed peak VL gradually increased during the early phase of the Beta wave, continuing to rise until about 200 days post-July 2020. Following this initial increase, observed peak VL declined slightly for about 50 days, before a second increase that lasted until around July 2021 (Day 350 post-July 1, 2020). In contrast, observed peak VL in Latin America remained relatively stable for the first 200 days of the study, with no VOC or VOI detected among the CoVPN 5001 participants enrolled in the region during this period. Around Day 200, observed peak VL began to rise from a median of 10^2.5^ copies/mL and reached a peak of ~10^4.6^ 100 days later (day 300 post-July 2020). This rise coincided with the emergence of the Gamma and Lambda variants.

The observation that increasing VLs were associated with viral “waves” was not universally observed in our dataset, however. Later time periods, such as the emergence of the Omicron variant (most of our cohort acquired the BA.1 or BA.2 variants), did not demonstrate a similar increase in observed peak VL coincident with the emergence of a new variant. During the Delta wave, for example, which persisted for about 200 days across all three regions until the onset of the Omicron wave, there was no significant increase in observed peak VL as observed with previous variants, such as Lambda or Gamma, which were later replaced by the Delta variant. Instead, observed peak VL consistently declined when Gamma and Lambda were replaced by Delta. We note that the Delta variant appeared to have been among the fastest to be replaced by Omicron, which had a lower observed peak VL compared to Delta.

Finally, we sought to assess whether temporal changes in observed peak VL coincided with changes to the characteristics of the enrolled population. While we detected small changes over time in some of these characteristics, such as BMI and age, these changes did not coincide with the temporal patterns of observed peak VL that we observed (Supplementary Figs. 3 and 4). Additionally, the day of enrollment post-symptom onset fluctuated over time in some countries, e.g., troughs of COVID days coincided with the onset of the Beta and Delta waves in South Africa (Supplementary Figs. 2 and 5). However, COVID day at enrollment was relatively uniform throughout the study in other countries, such as Peru and the USA. Taken together, these results suggest that VL changes dynamically over time and geography, independent of the assessed population host factors.

## Discussion

Based on the large CoVPN 5001 observational cohort study, we concluded that the infecting variant was associated with different viral kinetics. However, important host factors known to be associated with poor clinical outcomes had minimal associations with either observed peak VL or clinical viral shedding duration. The CoVPN 5001 cohort was unique in that it recruited over a period ranging from the early stages of the pandemic (mid-2020), where many participants experienced a primary SARS-CoV-2 acquisition, to the later stages of the pandemic encompassing the emergence of the Omicron variant (early 2022) in a population with some level of immunologic memory generated by both natural acquisition and vaccination. Observations from the CoVPN 5001 cohort therefore reflect both the historical emergence of COVID-19 and the beginning of the endemic Omicron era.

Based on previous reports, we had expected that more epidemiologically successful variants would have higher observed VLs and/or prolonged shedding compared to variants that were outcompeted and replaced during the COVID-19 pandemic^9,10^. Some of our observations were consistent with this prediction. For example, the Delta variant had both a higher observed peak VL and longer period of shedding when compared to the Beta variant. One rigorous study of viral kinetics between different variants was conducted using intensive serial sampling of NBA players^8^, but this population offers limited potential for observations with clinical and demographic variables due to a relatively homogenous population. Our study adds to an understanding of the differences in viral kinetics in the infecting variants across a heterogenous population and is consistent with previous observations that the Delta variant had higher observed peak VL and longer shedding duration than earlier variants. In keeping with this general concept that SARS-CoV-2 is adapting to the human host, all variants were estimated to have longer shedding duration and increased VL as compared to the reference strain. The analysis found that the Lambda and Delta variants were statistically significant after adjusting for host factors such as sex and age.

We also found differences between variants that were inconsistent with these predictions, as the variant strains with either the longest period of shedding or highest observed peak VL during our study differed from those that ultimately became predominant. For example, of all the variants captured in our cohort, the Lambda variant was associated with the longest clinical viral shedding and highest observed peak VL. This variant had limited spread outside of Latin America (where it no longer circulates) and is thus largely of historic interest^21,25^. Additionally, Omicron infections had viral kinetics relatively similar to those from the Reference variant. This observation despite two years of in-host adaptation may represent a combination of improved host control via immune memory in the face of improved viral mechanisms. Therefore, our data suggest that higher VL or longer clinical viral shedding duration, as assessed by PCR from nasal swabs during clinical infection, do not directly translate to increased viral fitness. Understanding the replication characteristics of variants that outcompete co-existing variants may be important for predicting the course of future respiratory viruses.

We found evidence of temporal trends in observed peak VL relative to the date when the epidemic started, but could not identify any clinical or viral factors, such as changes in age or BMI, that would consistently correlate with these trends. In contrast, we observed fluctuations in the COVID day (defined as the date of first symptom onset or positive COVID test) at enrollment over time, especially in some regions like Southern Africa, suggesting differences in care seeking. We propose that variations in observed peak VL represent a complex interplay between both the host behaviors and the pathogen. Several studies have used a fall in Ct value (as a surrogate for increasing VL) to estimate an epidemic status or potentially viral fitness^24,26^. For example, the initial outbreak of the Delta variant was associated with increases in observed peak VL^27^. However, our data suggest that direct interpretation of enhanced viral fitness from changes in Ct value should be done cautiously. For example, the Delta variant can encapsidate infectious viruses at a higher rate than other variants with the same input RNA^7^.

Our analysis, based on a large cohort representative of the spectrum of COVID-19 disease, found no meaningful associations of host demographic variables, including BMI, type-2 diabetes, and hypertension, with either observed peak VL or duration. Notably, HIV was not associated with prolonged clinical viral shedding in our cohort of participants living with HIV, where 91% reported taking antiretrovirals. A longitudinal study of outpatients in Southern Africa found that participants with a CD4 count lower than 200 cells/µL had prolonged shedding as compared to those with >200 cells/µL or individuals without HIV, but we did not have CD4 T cell count data on our participants and therefore cannot comment on the role of advanced HIV^28^.

Our findings describing a relatively weak relationship between host factors and viral kinetics of SARS-CoV-2 are like those from other respiratory pathogens. Prospective cohorts examining clinical viral shedding using twice weekly swabbing in a Southern African cohort found no association between host factors, including HIV (in adults) and shedding duration of respiratory syncytial virus^29^. Similarly, no association was observed with shedding duration in influenza^28^. The influence of host factors on clinical viral shedding should not be disregarded completely because, for example, children have prolonged shedding with both respiratory syncytial virus (RSV) and influenza compared to adults. Additionally, profoundly immunocompromised populations such as stem cell transplant recipients (not captured in our cohort) have increased shedding duration of many viral pathogens, including influenza^30^.

Strengths of our study include a large population representing multiple medical comorbidities, several geographic regions, and a standardized assay for measuring VL that includes viral sequencing. Additional technical strengths include a robust, validated process for specimen processing, and uniformity of virologic testing anchored to a validated copies/mL output. Our study utilized the same clinical infrastructure, validated end-point laboratories, and specimen pipelines that were used to support the licensure of several COVID-19 vaccines^31^. An additional strength is the method we used to account for the missingness of viral variant data. There is an intrinsic link between viral kinetics and the ability to assign a variant to a given clinical infection because assignment of variant status depends on the recovery of virus. Omitting VL data from analysis of clinical infections, from which a variant cannot be assigned based on sequencing data, could potentially lead to overrepresentation of more-fit variants. To address this concern, we implemented a method of predicting viral variants. We would recommend that studies that include SARS-CoV-2 variants utilize a similar approach.

We demonstrated in a large population with various medical comorbidities that the host factors do not associate with differences in virologic outcomes, whereas the infecting viral variant was strongly associated with differences in observed peak VL and the duration of clinical viral shedding. Our findings have important implications for the design of trials of antiviral interventions. Because we determined that host factors had minimal influence on viral outcomes, these trials should seek to enroll a broad population. As many of the host factors we assessed are strongly associated with poor clinical outcomes, our observations suggest there are additional factors independent of VL in the nasopharynx that mediate disease. Despite our observed associations between viral variant and VL and shedding duration, there remains substantial unexplained variability in viral kinetics during clinical infection with SARS-CoV-2. This suggests that additional factors influence virologic outcomes. Better understanding of these factors offers potential improvements for public health policy and insight into the SARS-CoV-2 pandemic to date.

A major limitation of our study is the lack of baseline measurements of immunity prior to SARS-CoV-2 acquisition. While we did not capture an effect of vaccination on a reduction in observed peak VL or clinical viral shedding, we cannot determine whether our participants had prior protective immunity. Based upon the timing of recruitment, our cohort likely includes a mix of individuals both immunologically naïve and those with pre-existing immunity. Vaccination status was captured on all participants, and most of the acquisitions in vaccinated participants occurred when Omicron became predominant. The lack of association between previous vaccination and either reductions in observed peak VL or shedding duration differs from some published studies^6^. Approximately half of our cohort received inactivated viral vaccines or viral vectors, whereas other cohorts more uniformly received mRNA vaccination. Despite increasing population-level exposure to SARS-CoV-2 as the pandemic progressed, neither observed peak VL nor shedding duration in the nasopharynx were substantially different between the Omicron and Reference strains in our cohort.

Another major limitation inherent to studies that enroll participants only after diagnosis (e.g., do not prospectively due viral surveillance) is substantial missingness of viral information from the early stages of infection. Prospective studies of SARS-CoV-2 have consistently demonstrated high VLs before the onset of symptoms, so we want to be clear that “observed viral peak” likely underestimates the maximum VL in the nasopharynx for many participants. Participants enrolled several days after symptom onset and after receiving a positive PCR test outside of the study. As a result, early VL measurements were unavailable for analysis and limiting our ability to study early viral kinetics. Our study may be representative of early observations of emerging variants, or interventional trials that require diagnostics prior to entry, and should be supplemented with prospective cohorts with periodic sampling^28,29^.

## Supplementary information

Transparent Peer Review file

Supplemental Information

Description of Additional Supplementary Files

Supplementary Data

## Data Availability

Data can be requested from the HIV Vaccine Trials Network (HVTN) and Statistical Center for HIV/AIDS Research and Prevention (SCHARP). In keeping with NIH policies on data accessibility, de-identified data is publicly available on Atlas (a system designed to have PHI protections): https://atlas.scharp.org/project/HVTN%20Public%20Data/begin.view. Source data for figures can be found using the Atlas link.

## Code availability

Code used to perform the analysis summarized in this paper can be accessed using the following link: https://github.com/HyrienLab/5001-paper.
