# Supplementary material for: Viral variant but not host factors associate with SARS-CoV-2 viral kinetics
Source: Commun Med (Lond). 2026 Apr 17;6:346. doi: 10.1038/s43856-026-01588-5 (PMC13272808; doi:10.1038/s43856-026-01588-5)
Supplement: Supplementary file 2 — Supplemental Information [file 43856_2026_1588_MOESM2_ESM.pdf]

A

	Roche Cobas		
**Fisher TaqPath**	Negative	Positive	Total
Negative	124	3	127
Positive	15	33	48
Total	139	36	175

B

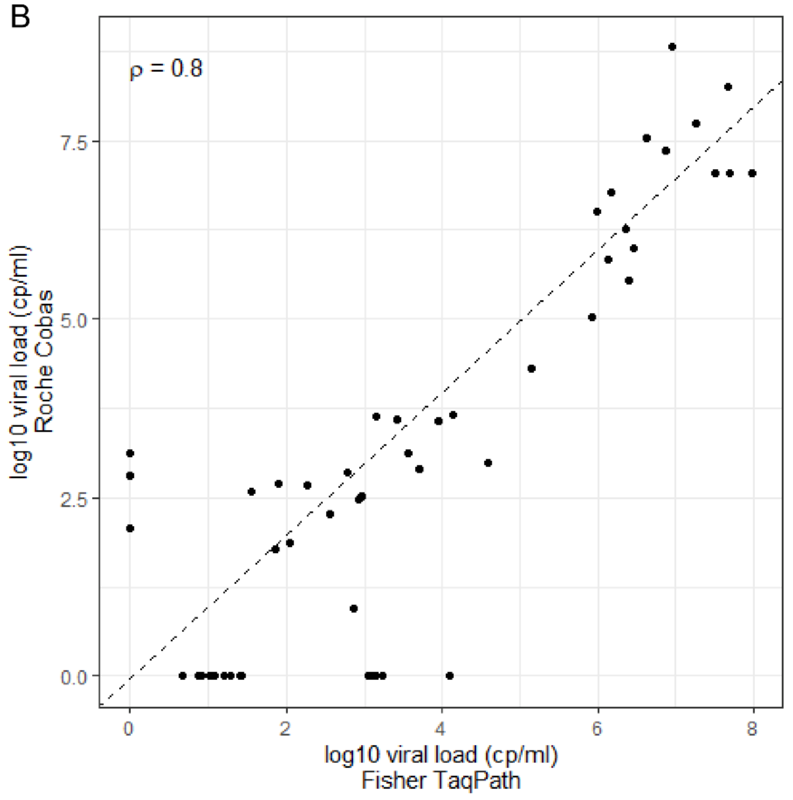

Supplemental Figure 1. Comparison of Roche Cobas and Fisher TaqPath RT-PCR testing. (A) Qualitative comparison of the 175 samples run on both assays. (B) Scatterplot of viral load (log10 copies/mL) for the N=51 samples that were qualitatively positive on at least one assay. Dashed line of identity and Spearman correlation are included for reference.

Supplemental Table 1. CoVPN 5001 Participant Demographics

Characteristic		Cohort			
		Group 1: Asymptomatic<br>98 (10.3%)	Group 2: Mild symptoms, not hospitalized<br>815 (85.5%)	Group 3: Hospitalized<br>40 (4.2%)	Total<br>953
Race	American Indian	0 (0.0%)	13 (1.6%)	0 (0.0%)	13 (1.4%)
	Asian	1 (1.0%)	6 (0.7%)	0 (0.0%)	7 (0.7%)
	Black	70 (71.4%)	341 (41.8%)	15 (37.5%)	426 (44.7%)
	Multiple Selected	1 (1.0%)	7 (0.9%)	0 (0.0%)	8 (0.8%)
	Native Hawaiian	0 (0.0%)	6 (0.7%)	0 (0.0%)	6 (0.6%)
	Other	19 (19.4%)	330 (40.5%)	14 (35.0%)	363 (38.1%)
	White	7 (7.1%)	112 (13.7%)	11 (27.5%)	130 (13.6%)
Hispanic-Latino/a	Hispanic or Latino	12 (12.2%)	398 (48.8%)	25 (62.5%)	435 (45.6%)
	Not Hispanic or Latino	86 (87.8%)	417 (51.2%)	15 (37.5%)	518 (54.4%)
Days from first symptom/+ test to enrollment	Mean (SD)	6.3 (2.76)	9.1 (3.33)	12.3 (10.14)	9.0 (3.96)
	Medium (25th, 75th %ile)	6.0 (4.0, 9.0)	9.0 (7.0, 11.0)	10.0 (7.0, 13.5)	9.0 (7.0, 11.0)
	Min - Max	1.0 - 12.0	2.0 - 36.0	5.0 - 58.0	1.0 - 58.0
Current smoke tobacco/cannabis	No	88 (89.8%)	721 (88.5%)	40 (100.0%)	849 (89.1%)
	Yes	10 (10.2%)	94 (11.5%)	0 (0.0%)	104 (10.9%)
Ever smoke tobacco/cannabis	No	81 (82.7%)	600 (73.6%)	34 (85.0%)	715 (75.0%)
	Yes	17 (17.3%)	215 (26.4%)	6 (15.0%)	238 (25.0%)
COPD/emphysema/asthma	No	97 (99.0%)	779 (95.6%)	37 (92.5%)	913 (95.8%)
	Yes	1 (1.0%)	36 (4.4%)	3 (7.5%)	40 (4.2%)
Congestive heart failure	No	96 (98.0%)	814 (99.9%)	35 (87.5%)	945 (99.2%)
	Yes	2 (2.0%)	1 (0.1%)	5 (12.5%)	8 (0.8%)
Chronic kidney disease	No	98 (100.0%)	810 (99.4%)	37 (92.5%)	945 (99.2%)
	Yes	0 (0.0%)	5 (0.6%)	3 (7.5%)	8 (0.8%)
Diabetes	No	96 (98.0%)	774 (95.0%)	34 (85.0%)	904 (94.9%)
	Yes	2 (2.0%)	41 (5.0%)	6 (15.0%)	49 (5.1%)
Renal disease/eye disease, peripheral neuropathy	No	98 (100.0%)	807 (99.0%)	38 (95.0%)	943 (99.0%)
	Yes	0 (0.0%)	8 (1.0%)	2 (5.0%)	10 (1.0%)
HIV	No	81 (82.7%)	753 (92.4%)	39 (97.5%)	873 (91.6%)
	Yes	17 (17.3%)	62 (7.6%)	1 (2.5%)	80 (8.4%)
Hypertension	No	87 (88.8%)	702 (86.1%)	28 (70.0%)	817 (85.7%)
	Yes	11 (11.2%)	113 (13.9%)	12 (30.0%)	136 (14.3%)
Immune system disorder	No	80 (81.6%)	739 (90.7%)	39 (97.5%)	858 (90.0%)
	Yes	18 (18.4%)	76 (9.3%)	1 (2.5%)	95 (10.0%)
Chronic corticosteroids	No	95 (96.9%)	749 (91.9%)	8 (20.0%)	852 (89.4%)
	Yes	3 (3.1%)	66 (8.1%)	32 (80.0%)	101 (10.6%)
Brief course of dexamethasone	No	98 (100.0%)	794 (97.4%)	13 (32.5%)	905 (95.0%)
	Yes	0 (0.0%)	21 (2.6%)	27 (67.5%)	48 (5.0%)
Positive pregnancy test	No	48 (49.0%)	436 (53.5%)	15 (37.5%)	499 (52.4%)
	Not Applicable	50 (51.0%)	375 (46.0%)	25 (62.5%)	450 (47.2%)
	Yes	0 (0.0%)	4 (0.5%)	0 (0.0%)	4 (0.4%)

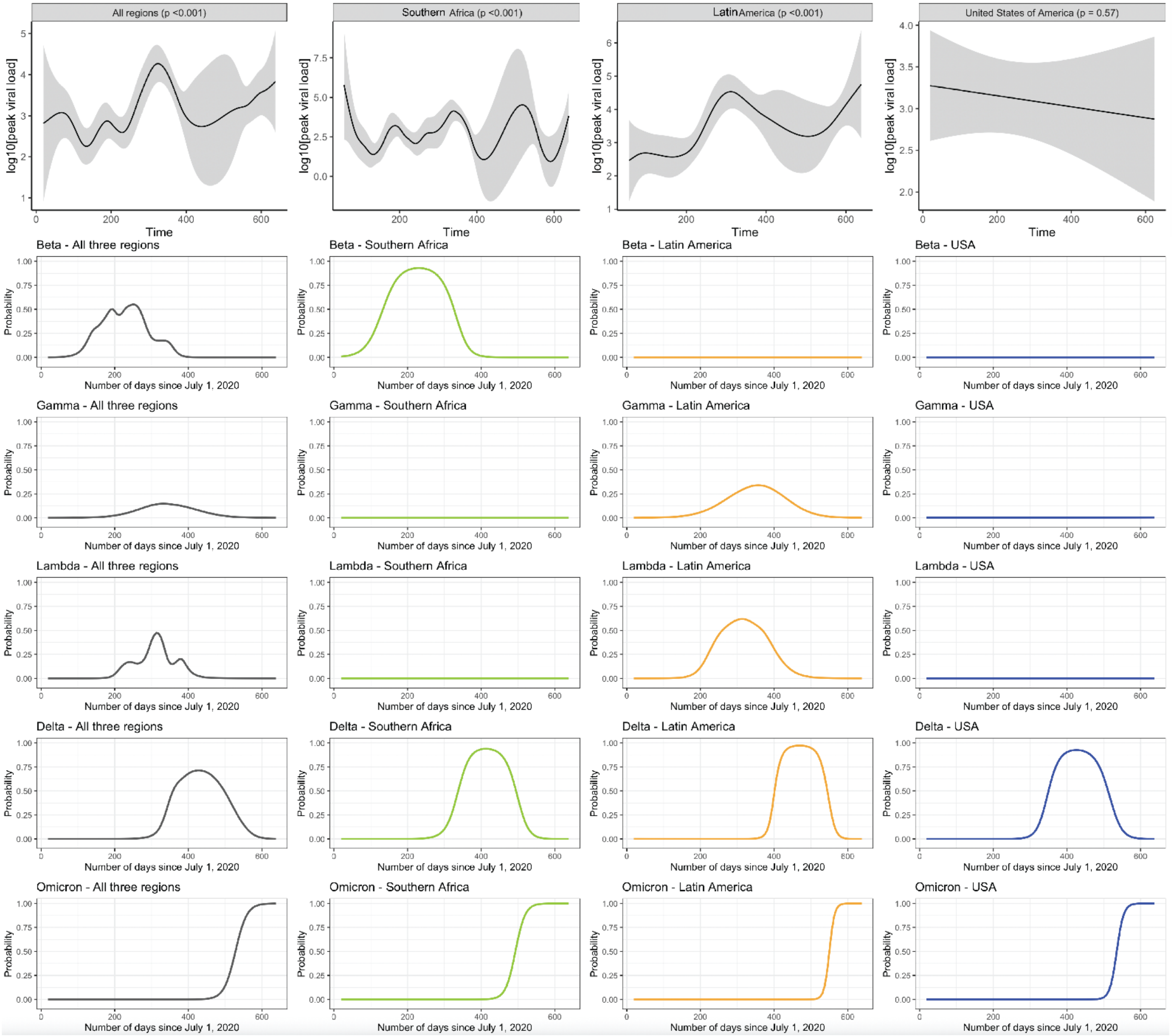

Supplemental Figure 2. Average log10 observed peak viral load over time compared to the probability of sampling a given variant over time, stratified by region in columns 2, 3, and 4. Column one represents the overall study cohort from all three regions. Row 1 represents the log10 observed peak viral load over time, inclusive of all variants sampled. Rows 2-6 represent the probability of sampling the indicated viral variant, stratified by region. N=953

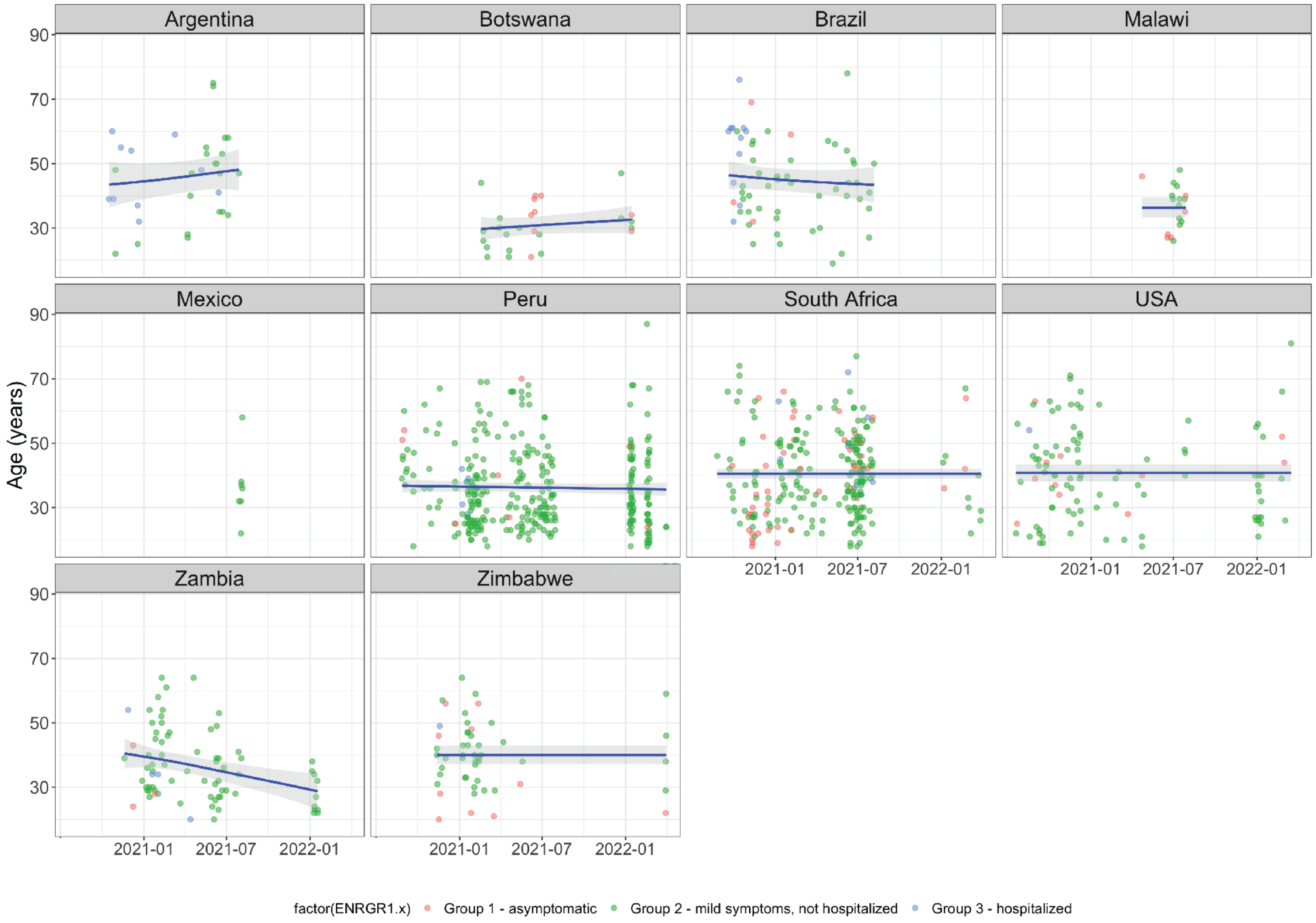

Supplemental Figure 3. Age of participants over time, by country. The line represents a point estimate of the average age of participants enrolled in the study at the date of enrollment, as estimated by general additive modelling (GAM). The shaded area associated with each region indicates the point-wise 95% confidence interval for age. N=953

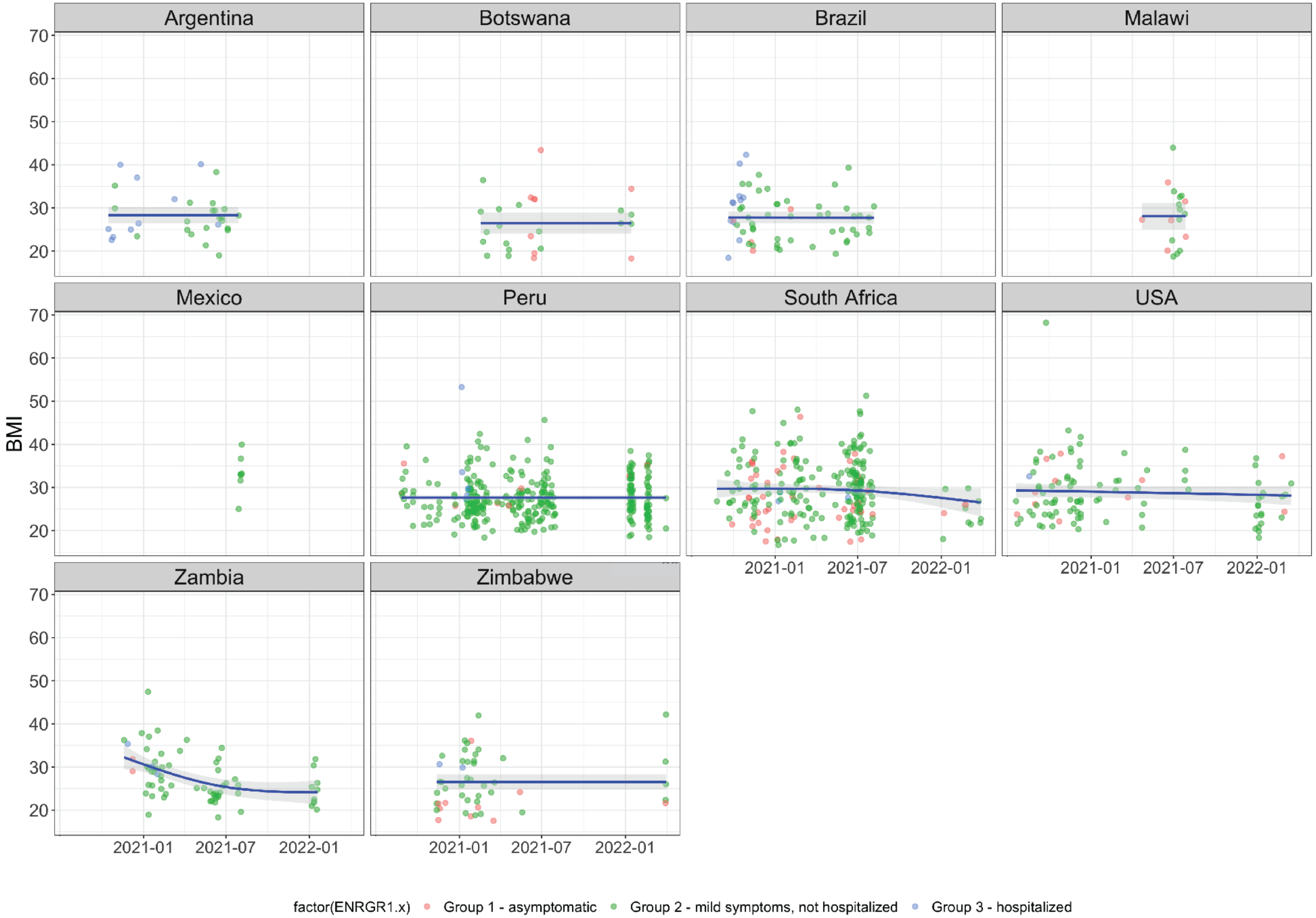

Supplemental Figure 4. BMI of participants over time, by country. The line represents a point estimate of the average COVID day of participants enrolled in the study at the date of enrollment, as estimated by general additive modeling (GAM). N=953

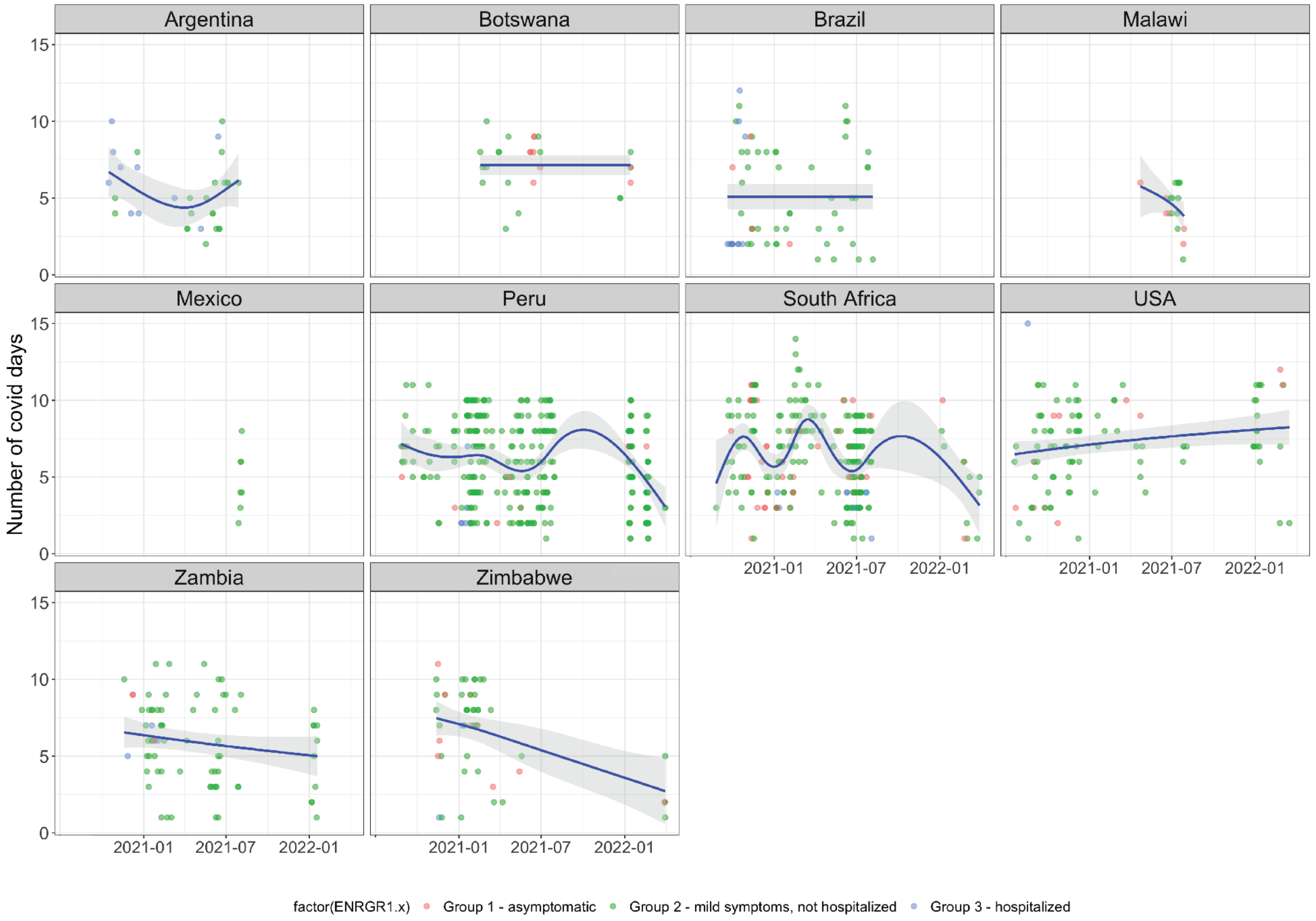

Supplemental Figure 5. Days since symptom onset or first positive test (if asymptomatic) over time ("COVID day"), by country. The line represents a point estimate of the average COVID day of participants enrolled in the study at the date of enrollment, as estimated by general additive modeling (GAM). N=953
