# Supplementary material for: Viral variant but not host factors associate with SARS-CoV-2 viral kinetics
Source: Commun Med (Lond). 2026 Apr 17;6:346. doi: 10.1038/s43856-026-01588-5 (PMC13272808; doi:10.1038/s43856-026-01588-5)
Supplement: Supplementary file 3 — Description of Additional Supplementary Files [file 43856_2026_1588_MOESM3_ESM.docx]

**Description of Additional Supplementary Files**

File name: Supplementary Data

Description: IRBs for the sites
