# Supplementary material for: Viral variant but not host factors associate with SARS-CoV-2 viral kinetics
Source: Commun Med (Lond). 2026 Apr 17;6:346. doi: 10.1038/s43856-026-01588-5 (PMC13272808; doi:10.1038/s43856-026-01588-5)
Supplement: Supplementary file 1 — Transparent Peer Review file [file 43856_2026_1588_MOESM1_ESM.pdf]

nature portfolio

# Peer Review File

## Viral Variant but Not Host Factors Associate with SARS-CoV-2 Viral Kinetics

Corresponding Author: Dr Ollivier Hyrien

**This file contains all reviewer reports in order by version, followed by all author rebuttals in order by version.**

Version 0:

Reviewer comments:

Reviewer #1

(Remarks to the Author)
The manuscript titled "SARS-CoV-2 Variant is Strongly Associated with Peak Viral Load and Shedding Duration with Minimal Impact from Host Factors: Results from the CoVPN 5001 Study" seeks to characterize the natural history of SARS-CoV-2 infection and investigate the relationship between viral shedding and clinical outcomes. However, it is marred by substantial methodological limitations, particularly in the analysis of viral shedding data. The most significant concern pertains to the definition of the viral load peak. The authors do not provide a clear explanation of how the peak was determined. If the authors merely considered the highest observed value, they may have overlooked the true peak (assuming there is only one). A viral kinetic model, such as the one proposed by Néant et al. (https://doi.org/10.1073/pnas.2017962118), would have been a more appropriate tool for estimating the time to and the height peak viral load as well as the duration of viral shedding. Without this critical step, it is impossible to accurately define the peak viral load or the duration of viral shedding, rendering the conclusions of the manuscript invalid.

Reviewer #2

(Remarks to the Author)
The authors estimate factors associated with peak viral load and viral shedding duration—key elements for virological assessment of infectious diseases and easily observable epidemiologically—through a large-scale study surpassing previously reported cohort studies. Their finding that differences in these characteristics by viral strain were not associated with the epidemic trends in a diverse population is highly significant for interpreting both virological and epidemiological assessments in future pandemics. Regarding host factors such as underlying conditions, since the estimates were based on relatively small classes, the lack of association warrants further discussion and should be interpreted alongside findings from other studies. Nonetheless, the study's methodology, analysis, and interpretation are well described, with clearly stated claims and limitations. This extensive and long-term investigation, encompassing a range of regions and population backgrounds while capturing the evolving nature of COVID-19 outbreaks, offers highly valuable insights. To further enhance the understanding of the observed results from the perspective of viral dynamics within the host, I would like to offer the following comments:

1. It seems necessary to clearly define what is meant by peak viral load here. In particular, providing information on how many days after the enrollment date (COVID days) the peak occurred would enable a better assessment of the validity of the analysis. The observed differences in peaks likely reflect differences in the progression of infection within the body, and caution is warranted when generalizing these as pure peak viral loads.
2. Would it be possible to show the time course of viral load for individual patients? In addition to differences between viral strains, it might be possible to discuss differences in disease severity from the perspective of changes in viral load over time. Moreover, making such data on viral load changes publicly available could contribute to more reproducible research and a better understanding of the evolution of infectious disease outbreaks.
3. Although the distribution of COVID days is presented, the large variability makes interpretation difficult. For instance, based on the relationship between detection date and changes in viral load, there have been attempts to reconstruct outbreak magnitude from the distribution of viral load at the time of detection in cross-sectional data (https://doi.org/10.1126/science.abh0635). Considering that the number of days since infection of patients included in the study may vary depending on the scale of an outbreak, is it possible to discuss the relationship between viral strains and peak viral load from the perspective of outbreak size for each strain?

Reviewer #3

(Remarks to the Author)
The paper by Hahn William et al. presents the findings of a large cohort study that examined the relationship between host factors and peak viral load and viral shedding. The study was conducted in a longitudinal cohort that was both demographically and clinically diverse, and it took into account the effects of viral variants. This observational study was conducted from July 2020 to July 2022, and enrolled 953 adults diagnosed with acute SARS-CoV-2. The authors determined that while host factors, including age, BMI, sex, medical comorbidities, and (surprisingly) HIV, demonstrated no significant associations with either peak viral load or viral shedding in the upper tract samples, viral variant demonstrated a significant association with peak viral load and viral shedding. Consequently, peak viral load and the duration of shedding may be interpreted as predictors of viral fitness.
The study is well written and designed. The data collated and analysed in this study are comprehensive and relate to a substantial cohort of persons. The methods employed are adequate to yield the anticipated results. The findings are of use in terms of public health response, as long as studies about the population dynamics of infective agents may provide valuable information for improving the efficiency of lockdown and quarantine protocols.
It would be beneficial to ascertain whether a quality assessment has been conducted between the laboratories involved in the VL analysis, and to explain how the performance of the test was assessed.
It is recommended that the methodology employed in the collection of the data be elucidated with greater precision, and that the individuals responsible for its collection be identified.
In addition, it is advised that the statistical analysis reported by Pellegrinelli et al. be consulted: 'A Flexible Regression Modelling Approach Applied to Observational Laboratory Virological Data Suggests That SARS-CoV-2 Load in Upper Respiratory Tract Samples Changes with SARS-CoV-2 Epidemiology' (2023).

Version 1:

Reviewer comments:

Reviewer #1

(Remarks to the Author)
please see the attached document

Reviewer #2

(Remarks to the Author)
The authors have responded to the peer review comments as sincerely as possible and have improved the manuscript accordingly. I have no further additional comments.

Reviewer #3

(Remarks to the Author)
The authors have taken the time to address all the reviewers' suggestions in a thorough manner. While some limitations remain, as they themselves acknowledge, the manuscript has improved as a result of the revisions.
I have no further comments to offer.

**Open Access** This Peer Review File is licensed under a Creative Commons Attribution 4.0 International License, which permits use, sharing, adaptation, distribution and reproduction in any medium or format, as long as you give appropriate credit to the original author(s) and the source, provide a link to the Creative Commons license, and indicate if changes were made.
In cases where reviewers are anonymous, credit should be given to 'Anonymous Referee' and the source.
The images or other third party material in this Peer Review File are included in the article's Creative Commons license, unless indicated otherwise in a credit line to the material. If material is not included in the article's Creative Commons license and your intended use is not permitted by statutory regulation or exceeds the permitted use, you will need to obtain permission directly from the copyright holder.
To view a copy of this license, visit https://creativecommons.org/licenses/by/4.0/

Reviewer #1 (Remarks to the Author):

The manuscript titled "SARS-CoV-2 Variant is Strongly Associated with Peak Viral Load and Shedding Duration with Minimal Impact from Host Factors: Results from the CoVPN 5001 Study" seeks to characterize the natural history of SARS-CoV-2 infection and investigate the relationship between viral shedding and clinical outcomes. However, it is marred by substantial methodological limitations, particularly in the analysis of viral shedding data. The most significant concern pertains to the definition of the viral load peak. The authors do not provide a clear explanation of how the peak was determined. If the authors merely considered the highest observed value, they may have overlooked the true peak (assuming there is only one). A viral kinetic model, such as the one proposed by Néant et al. (https://doi.org/10.1073/pnas.2017962118), would have been a more appropriate tool for estimating the time to and the height peak viral load as well as the duration of viral shedding. Without this critical step, it is impossible to accurately define the peak viral load or the duration of viral shedding, rendering the conclusions of the manuscript invalid.

**Response:** We thank the reviewer for their time and for their comments.

We have edited the manuscript to more carefully explain the methods used to describe the peak viral load. We have read the manuscript provided by the reviewer and cited it in our manuscript. We would caution somewhat against direct comparison between the two approaches as the cited reference describes a hospitalized cohort based upon biological and methodological considerations.  As many have demonstrated, hospitalized cohorts have distinct, prolonged shedding as compared to non-hospitalized cohorts. The larger concern is methodological as the study design in the cited paper was more able to capture early viral loads closest to symptom onset. Other cohorts using similar kinetic modeling in non-hospitalized patients (for example, NBA players locked in the “bubble”) has observations much closer temporally to the onset of infection because of the longitudinal nature of the sampling.

Our study design was more intended to mimic the practical study design of vaccine trials or antiviral interventions on less severe COVID, so our study only captured participants after symptom onset and a positive PCR. Practically, we could not obtain viral readouts as close to the timepoint the authors cite due to the nature of the study design. This is a limitation of the trial design and we have included a discussion of the limitation in the manuscript.  In order to fit these viral kinetic curves to our dataset, we would have to make strong assumptions regarding the viral load closer to the onset of symptoms. Our model adjusted for days since symptom onset. We anticipate that this adjusted peak viral load correlates with the biological peak viral load. In response to the reviewer’s comment, we have fleshed out this concept in the discussion.

Reviewer #2 (Remarks to the Author):

The authors estimate factors associated with peak viral load and viral shedding duration—key elements for virological assessment of infectious diseases and easily observable epidemiologically—through a large-scale study surpassing previously reported cohort studies. Their finding that differences in these characteristics by viral strain were not associated with the epidemic trends in a diverse population is highly significant for interpreting both virological and epidemiological assessments in future pandemics. Regarding host factors such as underlying

conditions, since the estimates were based on relatively small classes, the lack of association warrants further discussion and should be interpreted alongside findings from other studies. Nonetheless, the study's methodology, analysis, and interpretation are well described, with clearly stated claims and limitations. This extensive and long-term investigation, encompassing a range of regions and population backgrounds while capturing the evolving nature of COVID-19 outbreaks, offers highly valuable insights. To further enhance the understanding of the observed results from the perspective of viral dynamics within the host, I would like to offer the following comments:

1. It seems necessary to clearly define what is meant by peak viral load here. In particular, providing information on how many days after the enrollment date (COVID days) the peak occurred would enable a better assessment of the validity of the analysis. The observed differences in peaks likely reflect differences in the progression of infection within the body, and caution is warranted when generalizing these as pure peak viral loads.

**Response:** Thank you for this feedback. Please see the response to Reviewer # 1's comment above.

2. Would it be possible to show the time course of viral load for individual patients? In addition to differences between viral strains, it might be possible to discuss differences in disease severity from the perspective of changes in viral load over time. Moreover, making such data on viral load changes publicly available could contribute to more reproducible research and a better understanding of the evolution of infectious disease outbreaks.

**Response:** Thank you for this comment. With an "n" of greater than 950 participants, it is challenging to display the time course for each individual participant. We wholeheartedly agree with the review that a more in-depth secondary analysis could provide additional insights into infectious disease outbreaks. In keeping with NIH policies on data accessibility, we will make de-identified data publicly available on Atlas (a system designed to have some PHI protections).

3. Although the distribution of COVID days is presented, the large variability makes interpretation difficult. For instance, based on the relationship between detection date and changes in viral load, there have been attempts to reconstruct outbreak magnitude from the distribution of viral load at the time of detection in cross-sectional data (https://doi.org/10.1126/science.abh0635). Considering that the number of days since infection of patients included in the study may vary depending on the scale of an outbreak, is it possible to discuss the relationship between viral strains and peak viral load from the perspective of outbreak size for each strain?

**Response:** We thank the reviewer for the thoughtful feedback.

We had read the cited paper closely and attempted to reconstruct a similar analysis across both space and time. At some points in the pandemic in some locations (during the beta wave in South Africa, for example), our results mirror the findings from the Hay et al. paper. At other points (during the Omicron wave, for example) this relationship breaks down completely. We have revised our discussion to further highlight this complex relationship.

Reviewer #3 (Remarks to the Author):

The paper by Hahn William et al. presents the findings of a large cohort study that examined the relationship between host factors and peak viral load and viral shedding. The study was conducted in a longitudinal cohort that was both demographically and clinically diverse, and it took into account the effects of viral variants. This observational study was conducted from July 2020 to July 2022, and enrolled 953 adults diagnosed with acute SARS-CoV-2. The authors determined that while host factors, including age, BMI, sex, medical comorbidities, and (surprisingly) HIV, demonstrated no significant associations with either peak viral load or viral shedding in the upper tract samples, viral variant demonstrated a significant association with peak viral load and viral shedding. Consequently, peak viral load and the duration of shedding may be interpreted as predictors of viral fitness.
The study is well written and designed. The data collated and analysed in this study are comprehensive and relate to a substantial cohort of persons. The methods employed are adequate to yield the anticipated results. The findings are of use in terms of public health response, as long as studies about the population dynamics of infective agents may provide valuable information for improving the efficiency of lockdown and quarantine protocols.

*It would be beneficial to ascertain whether a quality assessment has been conducted between the laboratories involved in the VL analysis, and to explain how the performance of the test was assessed.*

**Response**: All laboratory analysis was conducted using validated labs intended to support the licensure of vaccines for COVID-19. All measurements were in copies/mL (not CT values) with each assay running standards concurrently with each sample to generate this value. To further support our conclusions that the measurements of viral load were technically robust, repeat experiments were conducted with a subset of samples sent to both end-point labs. There was 90% agreement qualitatively (presence or absence of SARS-CoV2). Of the specimens positive at both labs, the quantitative results were highly correlated (Spearman correlation of 0.8). We have added some of this detail to the methods section.

*It is recommended that the methodology employed in the collection of the data be elucidated with greater precision, and that the individuals responsible for its collection be identified.*

**Response**: We have added some additional detail about the data collection and QC process to the manuscript.

*In addition, it is advised that the statistical analysis reported by Pellegrinelli et al. be consulted: 'A Flexible Regression Modelling Approach Applied to Observational Laboratory Virological Data Suggests That SARS-CoV-2 Load in Upper Respiratory Tract Samples Changes with SARS-CoV-2 Epidemiology' (2023).*

**Response:** We thank the reviewer for their kind comments and the provided reference. We have consulted this reference and updated the methods section to clarify that our approach was generally consistent with the approach described by Pellegrini et al. We have also included this citation in the revised manuscript.

We appreciate the reviewer's comments and agree that our study did not capture the true peak viral load. Accordingly, our findings pertain specifically to the observed peak viral load. We have revised the manuscript throughout to clarify this distinction. We also acknowledge that, like many real-world cohorts, our study design did not allow for capturing the earliest viral dynamics immediately following exposure. In response to the reviewer's comment, we have updated our terminology to refer to "clinically" relevant shedding duration, as this more accurately reflects the timeframe beginning at symptom onset or first positive clinical test, key moments when patients typically present for care and become eligible for intervention. This framing aligns our analysis with the practical realities of clinical trial enrollment and therapeutic decision-making.

For example, in the introduction we write the following: We determine that host characteristics and clinical variables exhibited minimal associations with both observed peak VL (i.e., the practical maximum observed viral load in a clinical trial where enrollment occurs after the onset of symptoms) and clinical viral shedding duration (the practical time of viral shedding after diagnosis), suggesting that these variables may not be useful in tailoring public health interventions targeting populations with potentially higher transmission risks.

We have also clarified peak viral load as "observed peak viral load" and "viral shedding" as "clinical viral shedding" throughout the text and in figures.

As with many other studies, including Ke et al, we did not perform serial testing in asymptomatic individuals and therefore did not capture the earliest phase of viral replication. While Ke et al. anchored some of their findings to cohorts with early, frequent testing (e.g., NBA players), those cohorts were composed of relatively homogeneous populations. In contrast, our cohort is highly diverse, and one of our primary objectives was to understand how clinical variables influence clinically relevant viral trajectories. As such, we could not assume that viral kinetics observed in the NBA cohort would generalize to our population. We explicitly acknowledge this as a limitation in the discussion, while emphasizing that our study design reflects real-world clinical contexts where early asymptomatic testing is rarely feasible.

If we assume that the model based peak viral load predicts the actual peak viral load early in infection, the absence of correlation between the observed peak and the peal predicted by the model in would suggest that any association between any covariates and the observed peak viral load would not identify a correlation between the covariate and the actual peak viral load. To test this, we extracted the observed peak from the predicted peak (see figure below). Since there is a strongly positive correlation between both the predicted viral and predicted viral shedding by the model in Ke at al., and the observed peak and shedding duration measured in NBA players, we feel that our conclusions regarding the effects of covariates in our model are likely to be robust.

Due to the enrollment design of the 5001 study, we were unable to use actual peak viral load or true shedding duration to identify factors associated with viral kinetics. Instead, we relied on observed peak viral load and clinical viral shedding duration as proxies. This approach would be valid if these observed measures were positively correlated with their true counterparts. To assess this, we extracted peak viral load estimates from the fitted models published in Ke et al. and correlated them with the observed peak viral load and clinical shedding duration used in our

analysis (see figure below). The results showed strong positive correlations in both cases, with Pearson correlation coefficients of 0.77 and 0.81, and corresponding p-values of 0.027 and 0.015, respectively. Assuming that dynamic models of viral kinetics provide accurate estimates of true peak viral load and shedding duration, these findings support the use of observed measures as reasonable surrogates. Therefore, we believe our conclusions regarding covariate effects are likely to be robust.

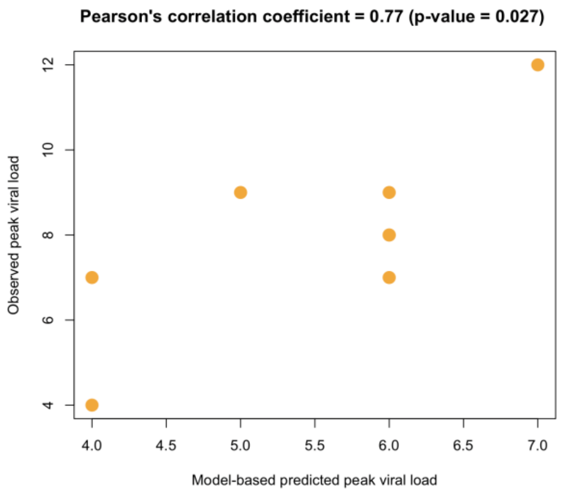


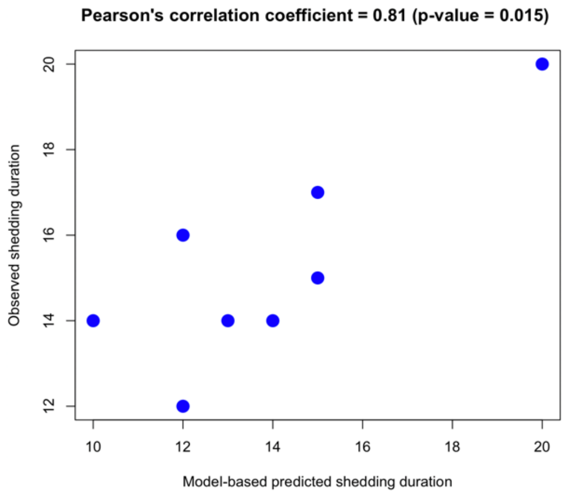


Our analysis remains valuable even if the observed peak viral load in our cohort does not perfectly capture the true biological peak. In fact, the observed peak is likely more clinically relevant, as it reflects the timing of presentation to care and enrollment in interventional trials, precisely when therapeutic decisions are made. Moreover, host factors are unlikely to substantially influence either the observed peak or the clinically relevant shedding duration. This reduces the risk that population composition confounds the interpretation of intervention effects. By focusing on metrics that align with real-world clinical decision points, our approach enhances the applicability and robustness of our findings in evaluating the impact of immunomodulatory or antiviral agents.
